# BiGART 2025 – the 25th Acta Oncologica Symposium

**DOI:** 10.2340/1651-226X.2025.44981

**Published:** 2025-11-26

**Authors:** Jens Overgaard, Morten Høyer, Birgitte Vrou Offersen, Karen-Lise Garm Spindler, Jesper Grau Eriksen

**Affiliations:** a Department of Experimental Clinical Oncology, Aarhus University Hospital, Aarhus, Denmark; b Danish Centre for Particle Therapy, Aarhus University Hospital, Aarhus, Denmark

**Keywords:** BiGART 2025, Acta Oncologica Symposium

The BiGART 2025 symposium was an open integrated Scandinavian radiotherapy meeting devoted to the development of new principles for radiotherapy. These include biological basis, the identification of tumors and tissues with contemporary imaging, and the ability to deliver radiotherapy in a precise and flexible three-dimensional way, all delivered in a real-time interactive, adjustable, and controlled fashion. BiGART 2025 is the 10^th^ in this series of Biological image Guided Adoptive RadioTherapy meetings [1–9], and as most of its predecessors, this was again hosted at the Helnan Marselis Hotel at the Bay of Aarhus in beautiful early summer surroundings.

The BiGART symposia are unique in the sense that they bring together people who are interested in modern radiotherapy with a focus on the clinical delivery and outcome, and discuss it in one plenary room with all participants able to give their contributions, both as presentations and in the often lively discussions. Such open integrated platform with a mixture of clinicians, diagnosticians, physicists, and biologists has given the meetings a special atmosphere, which, in turn, creates beneficial long-lasting collaborations, and it is definitely a needed type of platform for securing integrated understanding and exchange of ideas.

**Figure 1:**
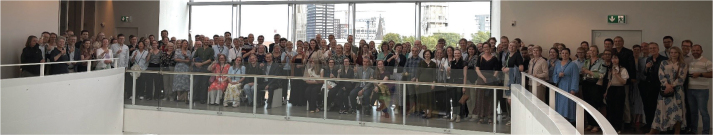
The BiGART 2025 participants.

This year, 155 participants from 11 countries (Figure 1) were present and presented 135 contributions in the form of overview presentations, original contributions, flash talks, and integrated poster discussions. Thus, almost all participants were actively contributing in one form of presentation or another in addition to the many discussions. The 2025 symposium also marked a change in the organizing team, which, in the past, has been led by Cai Grau, Morten Høyer, and Jens Overgaard, but who now have handled the baton to the next generation: Birgitte Vrou Offersen, Karen-Lise Spindler, and Jesper Grau Eriksen now being responsible for the current and future BiGART activities.

BiGART 2025 was the 10^th^ Acta Oncologica Symposium in the series, and especially this year, the program was influenced by contributions related to AI and our attempts to integrate this new knowledge and tools in an optimal and useful way for daily practice. This was illustrated during the sessions, which, in addition, also realized the important need for a more individual oriented radiotherapy, not only by the adaptive delivery but also by taking into consideration the importance of the patients’ needs and conditions, especially the individual biological sensitivity of the tumor and surrounding tissue. Radiotherapy has gained a significant advantage of modern technology and access to computer power, which influence biology, imaging, and treatment delivery. So, all brought together, radiotherapy has probably more than other cancer disciplines, taken full advantage of this booming knowledge.

A weaker and least developed aspect of this development, however, is (the lack of) our understanding of biological individualization of tumors and tissues and their consequential impact on the given treatment. Whereas the treatment delivery and dose planning are fitted to the individual patients, the target and given dose to most of the frequent cancer diseases is very much based on a ‘one size fits all’ concept, and only little biological individualization is applied in the radiotherapy of, for example, cancer of the breast, lung, prostate, or squamous cell carcinomas, where radiotherapy has its main impact.

We have spent the last one or two decades with a sharp focus on treatment-related morbidity, with a strong focus on treatment delivery and associated optimal treatment planning and precision to avoid normal tissue damage. An activity clearly expressed by the four large proton centers, which has been established in the Scandinavian countries during this period.

During the same time, we have paid very limited attention to optimizing the treatment of the tumors, which is after all the one and only reason for delivery of any kind of radiotherapy to the patients. We must never forget that any curative intended cancer treatment, which does not result in a successful tumor control, is an most unwanted result, only leaving the patient with the side-effects of the treatment and a reduced possibility for an alternative therapeutic strategy. It is a key problem that we have become almost ignorant when it comes to identify and optimize the treatment of tumors, which are our prime and only target. Although securing of tumor control has been a constant challenge through the history of radiotherapy, it is ironic that when we now have reached an era with the biological and technical tools to explore the options of personal directed radiotherapy, then we are largely ignoring the task. With our freedom of research also comes an obligation, and the radiotherapeutic community must always strive to secure that any (curative) treatment should fulfill its goal; to control the tumor within the target. Therefore, the quest for the individual patient’s optimal tumor therapy must always be our foremost and driving task.

Today this is not so. We have very limited focus on the individual biological optimization of tumor treatment taking issues such as stem cells, hypoxia, repopulation, and individual sensitivity into consideration together with the patient’s general status [10–15]. The few recent attempts have, for example, been seen in the treatment of human papilloma virus (HPV) positive head and neck squamous cell carcinomas in the form of de-escalation of the treatment [16], again focusing on reducing morbidity rather than ensuring a good cancer control outcome. Another example is the Danish Breast Cancer Group (DBCG) Natural randomized trial (NCT03646955) exploring the indication, if any, for postoperative breast cancer radiotherapy. In the present days, the increasing use of hypofractionation has clearly been established to secure the patients comfort, although it is evident that such treatment from a biological point of view is delivering a relatively poorer therapeutical ratio [17], than what can be achieved by more conventional or hyperfractionated radiotherapy [18].

Ironically, the highest citations from Acta Oncologica are from a special issue on ‘Fractionation in Radiotherapy’ [19], which was organized and published in 1988. It was during the heyday of clinical fractionation research where we were about to understand the relationship between dose, number, and size of fractions and overall treatment time and the impact on tumors, and early and late normal tissue reactions. This knowledge, which is expressed in the alpha/beta model and has been a beneficial biological guide of radiotherapy during the 1990s and 2000s, has since been thrown overboard. Not because it is not valid or that human biology has changed but based on the philosophy that with modern technology, we can hit the target better. Consequently, we can avoid unneeded critical normal tissue and deliver our treatment in a simpler way using fewer and larger fractions. All this may well be true, but we must still remember that the human body and its response to ionizing radiation has not changed over the years. So, while it may be easier for the patients (and society) to undergo treatment with fewer fractions, it is still clear that the therapeutic ratio of hypofractionated treatment is inferior to the opposite, namely, a treatment delivered with many small fractions, such as hyperfractionated accelerated radiotherapy [20, 21]. Today’s practice is therefore just a different balance on the weight where a more precise delivery of radiotherapy is counterweighting a slightly poorer biology.

This is not true progress, and we are still in a situation where much can be gained in the form of optimal biological delivery of radiotherapy. We do not need to sell out of our past knowledge but rather integrate it with modern technological treatment delivery, given with a more individual biological approach.

The BiGART meetings in Scandinavia have helped to maintain the strong interest for radiotherapy in the region. There is no other place on the globe, which has witnessed a more profound investment and activity in modern radiotherapy equipment [22]. The 26 million Nordic people have now access to four proton facilities in addition to the experimental Boron Neutron Capture Therapy (BNCT) unit in Finland [23]. In general, the technological standard at the Scandinavian radiotherapy departments is very high, and since the countries are also characterized by free national healthcare systems, it implies that the Nordic region probably is the place in the world where patients have the best access to high technological modern radiotherapy delivered according to national evidence-based guidelines. The BiGART symposia strive to maintain this development, which not only is a reflection of the current status but also is built on the long-term traditions for academic and industrial collaboration in the Scandinavian countries. It goes way back in our history, with the development of the Betatron by the Norwegian physicist Rolf Widerøe [24], and later the development of modern linear accelerators, the multileaf collimator, and the basis for Intensity-Modulated Radiation Therapy (IMRT) with a strong Swedish involvement not least by Hans Svensson and Anders Brahme [25, 26] and their activities in Stockholm and Umeå. Today, IMRT has become the standard routine technology for everyday radiotherapy. In parallel, the associated early computerized dosimetry was developed by a joint Scandinavian university project [27]. Following this, stereotactic body radiation therapy (SBRT) was also developed in Sweden [28, 29], and all have used Acta Oncologica as their vehicle for publication. This long-lasting technological invention was brought into the integrated national healthcare systems in Scandinavia, which subsequent became the prime base for radiotherapy research [30, 31] with large clinical trials developing the evidence for clinical radiotherapy, not least through the Danish Multidisciplinary Cancer Groups (DMCG) (https://www.dmcg.dk) [32]. Again, this took benefit from the collaborative infrastructure, which existed in this part of the world where most patients are included in evidence generating clinical trials, and as such, the Scandinavian countries have influenced and contributed very significantly to international databases and guidelines, exemplified by the repeated large Swedish studies of technology assessment in radiotherapy and healthcare [33, 34].

Much of this activity has taken place through publications in Acta Oncologica, which has the unique situation of being an independent journal owned by its own scientific foundation with a background in the Nordic Oncological Societies. The foundation has been rather successful and has generated an income, which, in addition to running the journal also, is used to enhance the scientific knowledge in the Scandinavian countries in the form of Acta Oncologica symposia. This activity has been in place since the first symposium in 1999 [35], and the current BiGART 2025 symposium has the honor of being the jubilee 25^th^ Acta Oncologica symposium. The 40 publications originated from BiGART, which can be found in the special compilation of papers in Acta Oncologica, reflect the activities and contents of the BiGART 2025 meeting and what goes on in the Scandinavian area today within this field. It clearly indicates that the joint activities in radiology and radiotherapy were instituted by Gösta Forssell who together with the Nordic Radiology Societies founded Acta in 1921 [36], and today, more than a hundred years later, the journal is still very much alive.

Although the focus and activity are swinging from one area and country to another, and among the Scandinavian countries also having a difference in the weighting of the various activities, the whole region has a strong and stable tradition for maintaining and optimizing the field of radiotherapy.

The organizers and Acta Oncologica wish to thank the contributors, participants, and our dedicated staff and sponsors. We are looking forward to the next BiGART Acta Oncologica symposium in a few years of time.
